# Beyond the logo: Sanitas' experience in advancing inclusive sports mindset through Paralympic sponsorship

**DOI:** 10.3389/fspor.2026.1718212

**Published:** 2026-02-12

**Authors:** Olga Kolotouchkina, Luis Leardy, Javier Pérez-Tejero

**Affiliations:** 1 Department of Applied Communication Studies, Institute of Knowledge Technology, Complutense University of Madrid, Madrid, Spain; 2 Faculty of Media and Communication Sciences, Complutense University of Madrid, Madrid, Spain; 3 Spanish Paralympic Committee, Madrid, Spain; 4 “Fundación Sanitas” Chair for Inclusive Sport Studies (CEDI), Department of Health and Human Performance, Faculty of Physical Activity and Sport Sciences—INEF, Universidad Politécnica de Madrid, Madrid, Spain

**Keywords:** inclusive games, media representation of disability, Paralympic Games, people with disabilities, the Spanish Paralympic Committee

## Abstract

**Introduction:**

Sponsorship is an essential element of the sports ecosystem, enabling powerful and authentic engagement between brands, athletes, and teams. While sponsorship has been widely researched in the context of high-profile mainstream sports and global sporting events, the unique challenges, nuances, and best practices of Paralympic sponsorship remain significantly underexplored. The institutional framework of Paralympic Sponsorship in Spain launched in 2005 has enabled the development of remarkable partnerships between the Spanish Paralympic Committee and the corporate sponsors. Sanitas, a Spanish health insurance company and part of the BUPA Group, has transformed the format of Paralympic sponsorship into a holistic inclusive sports ecosystem.

**Methods:**

This case study aimed at systematizing the innovative sponsorship approach of Sanitas, adopts an exploratory, interpretive research approach, drawing on multiple sources: desk research of the Spanish Paralympic Committee, Sanitas Foundation and BUPA; semi-structured interview with the Sanitas Chief Sustainability and Corporate Affairs Officer and General Manager of Sanitas Foundation; as well as the insider research perspective of two authors, who are professionally engaged in the activities of the case under study. The second author has been leading the corporate communications area of the Spanish Paralympic Committee (SPC) since 2005. The third author has been leading the Sanitas Foundation Chair for Inclusive Sports Studies since 2017.

**Results:**

The review of Sanitas’ long-term partnership with the SPC illustrates a consistent, strategically grounded approach to Paralympic sponsorship, while also provides actionable insights for brand managers, Paralympic teams, and athletes navigating this promising but complex sponsorship landscape. The historical evolution of Sanitas’ involvement with the SPC shows a transformative journey from an initial focus on financial and healthcare support, to the creation of multi-stakeholder alliances, academic research and the development of initiatives to promote an inclusive social mindset.

**Discussion:**

While the Paralympic movement, and Paralympic sponsorship in particular, still face persistent challenges, including structural power imbalances and the lack of objective representation of athletes with disabilities, innovative and disruptive Paralympic sponsorship strategies may challenge the traditional divide between athletes with and without disabilities, redefine the meaning of inclusion in sport, and act as powerful drivers of social transformation.

## 1 Introduction

Sponsorship is an essential element of the sports ecosystem, enabling powerful and authentic engagement between brands, athletes, and teams (1–3). The commercial and social value of sport properties increasingly draws interest from brands and corporations in becoming sports sponsors. The sponsorship practice provides athletes, teams or sports events with financial aid or other specific support, while sponsors, in exchange, enhance their media exposure and communication impact through association with remarkable sports experiences and outstanding achievements. An effective sponsorship management underpinned by organizational capabilities, consistency, congruence and symbiosis among the sport property and the sponsor enables the creation of long-term shared value as well as generate significant social impact (4, 5).

While sponsorship has been extensively researched in the context of high-profile mainstream sports and global sporting events (6–11), scholarship addressing Paralympic sponsorship is still scarce and mainly focused on persistent inequities in marketing representation and media attention directed toward the Paralympic Games and their athletes. Burton, Naraine and Scott (12, p. 13) highlight that “the Paralympic Movement continues to be devalued as a sport property in the eyes of corporate partners”. Similarly, Legg and Dottori (13, p. 266) argue that the marketing of the Paralympic sport in general, and the Paralympic sponsorship in particular, is yet “in a relatively nascent stage of development”. Misener et al. (14) further note that the status and magnitude of the commercial value of the Paralympic Games is significantly constrained compared to the Olympics or other major sporting events, limiting the engagement of potential sponsors to support Paralympic athletes and teams.

Since the London 2012 Paralympic Games redefined the visibility and representation of Paralympic athletes (15–17), brands have shown growing interest in commercial partnerships with them. Jackson-Brown (18) reveals the increasing utilization of Paralympic athletes by powerful national and global brands to give signification to the Paralympic Games and to market new meanings of social inclusion. Among the most frequently featured elite athletes' profiles in advertising and communication campaigns by brands seeking to endorse their products and services with inspirational narratives of extraordinary achievements and inclusion are the technologically enhanced Paralympians using advanced prosthetics and racing wheelchairs (19–21).

Enhanced live broadcasting of the Games not only increases sponsor exposure but also generates valuable marketing opportunities for products and services (12). For instance, the Paris 2024 Paralympic Games set a new milestone in coverage, with more than 763 million hours of live broadcasting, an 83% increase compared to Tokyo 2020 and a 40% rise in global live audiences (22). Beyond the sporting arena, Paralympic athletes are also becoming increasingly relevant subjects within the logic of visual culture through their participation in popular television shows, serving as brand ambassadors in advertising campaigns, featuring in lifestyle magazines, and building influence as social media content creators (21).

A deeper understanding of the underlying logic and dynamics that drive brands to engage with the Paralympic Movement and to sponsor elite Paralympic teams and athletes is needed. Such a nuanced perspective can enrich sponsorship and sports marketing scholarship and practice with valuable insights into the associated complexities, emerging challenges, and potential impacts. These insights can also help address a space that remains underexplored compared with mainstream sports sponsorship and provide guidance for brands that have not yet engaged in Paralympic sponsorship but are seeking meaningful ways to align with values of inclusion, diversity, and social responsibility.

This study seeks to address this gap by examining the Paralympic sponsorship experience of Sanitas, a Spanish health insurance company and part of the BUPA Group. It is guided by the following research question: *What are the key strategic dimensions and action lines of the Paralympic Sponsorship model developed by Sanitas?* Sanitas joined the Paralympic Movement in 2007 through a partnership agreement with the SPC. What began as a traditional sponsorship initiative has since evolved into a holistic and inclusive sports model. The contribution of Sanitas to promoting inclusive sports practiced jointly by people with and without disabilities, is also a central focus of this study. As the International Paralympic Committee (IPC) identifies Paralympic sport as a key driver of a more inclusive society, the awareness-building and practice of inclusive sport promoted through Sanitas' Paralympic sponsorship are of particular relevance to this research.

Following this Introduction, we first review the scholarship on Paralympic sponsorship from the lens of the status of the Paralympic Games, the prevailing models of disability representation and narratives, and the role of Paralympic athletes as new subjects of commercial communication logic. After describing our research method, we present the case study of Sanitas, the Paralympic Sponsor in Spain since 2007. The paper concludes by discussing the theoretical and managerial implications of our research for scholars and practitioners of Paralympic sponsorship.

## 2 Theoretical framework

### 2.1 The context of the Paralympic sponsorship within the Paralympic Movement

The singularity of Paralympic sponsorship is strongly shaped by the historical evolution of the partnership between the International Olympic Committee (IOC) and the IPC, the processes governing host city selection, and the structure of The Olympic Partner (TOP) program, which is the principal sponsorship channel for both the Olympic and Paralympic Games. Legg et al. (23) emphasize the historical power imbalance between the IOC and IPC as a key factor underlying persistent inequalities in media visibility, global appeal, and social impact between the two events.

While the Paralympic Games have become the foremost international platform for showcasing athletes with disabilities and advancing their empowerment through sport with an ever-expanding number of participating countries, increasing gender parity, and extensive live broadcasting of competitions (15, 16, 24), they are still too often framed as secondary to the Olympics. Even joint initiatives such as the TOP program, which provides global sponsors with exclusive marketing rights to both Games, have not corrected this imbalance. Becker (25), for instance, highlights striking disparities in funding allocation: the IPC received approximately USD 14 million for marketing and broadcasting rights, compared to an estimated USD 3 billion directed toward the Olympics. This asymmetry also reflects broader debates in global sport governance and the evolution of mediated sports experience into a global entertainment phenomenon. The IOC's dominant role in driving the commercial and symbolic value of sports mega-events illustrates how institutional hierarchies perpetuate unequal recognition of Paralympic sport. As scholars have argued, this dynamic reveals a tension between the rhetoric of inclusion and equality promoted by the Olympic Movement and the material realities of resource distribution, branding power, and media exposure (17, 24). Furthermore, while mediated sports consumption exponentially increases on broadcasting, streaming and social media platforms (26–28), media visibility and consumption of Paralympic sport experiences is limited (20, 29).

The catalytic role of the Paralympic Games in bringing under the global spotlight the experience of disability as well as its underlying challenges and barriers are increasingly acknowledged (30, 31). The enhanced media visibility of the achievements of para-athletes during the Paralympic global event, both on the sports arena and in their personal life, contributes to effectively addressing persisting stereotypes of disability representation and embedded social inequalities experienced by people with disabilities (17, 32, 89, 90). Among the main impacts of the Paralympic Sponsorship, Legg and Dottori (13) highlight a critical combination of corporate associations with remarkable athletic performance and strong social commitment. In addition to the enhanced corporate values of diversity, equality and inclusion, Macdougall, Nguyen and Karg (5) also draw attention to the impact of Paralympic sponsorship on internal engagement and motivation of employees and key stakeholders through inspiring experiences with athletes with disabilities. The transformational impact of Paralympic sponsorship on promoting and increasing the social visibility of Paralympic sport, on raising global awareness of the critical challenges faced by people with disabilities, and on aligning brand performance with values of equality, diversity, and inclusion (14, 33), makes Paralympic sponsorship a powerful corporate asset for generating meaningful connections with consumers and enhancing a brand's positive social impact (34).

In parallel, the increasing presence of young para-athletes competing in popular sports categories in the Games (35, 36) and citizen engagement strategies launched by the Games host cities make the event particularly attractive and fun for younger audiences and contribute to transforming cultural schemas of disability (37–40).

Furthermore, the critical impact of Paralympic sponsorship in providing essential financial support to enable access to sports facilities, adaptive transport, specialized healthcare services, dedicated coaching and the equipment required for athletes with disabilities to compete at an elite level is receiving increasing scholarly attention. McPherson et al. (32) highlight that costs of the required special equipment are not affordable with the usual help from families, charities or willingly given communities. In fact, Howe (41) notes that while high-end mobility technologies are central to elite Paralympic performance, they are also exclusionary as the costs of racing wheelchairs are over £5,000 and of ergonomically designed prosthesis up to £20,000, preventing many potential athletes from ever reaching elite competition.

### 2.2 Paralympic sponsorship and the critical challenges of disability representation

The power asymmetry of the Paralympic Games raises important questions about the representation of disability and inclusion of the Paralympic movement. While a global understanding of disability and the visibility of Paralympic movement remain limited, Paralympic sponsorship practice and related brand storytelling increasingly influence how disability is represented and perceived in mainstream culture through advertising, corporate communications, and social media. Despite the Paralympics becoming a global visibility platform for athletes with disabilities, this visibility is still mediated by commercial logic and cultural narratives that often frame disability through limited or stereotypical lenses (19, 20, 29, 32, 42).

A notable example is Toyota's Super Bowl 2021 commercial featuring American Paralympic swimmer Jessica Long. The ad, which dramatized her personal journey to Paralympic success, was widely celebrated as one of the event's most powerful and moving commercials (43, 44). However, it also sparked criticism from disability activists, who launched the #ToyotaWeDisapprove campaign, arguing that the ad perpetuated ableism by framing disability through inspiration porn (45). This case reveals the complexity of disability narratives that may resonate with non-disabled audiences, but in parallel be detrimental to disability-rights movements (20).

Among the most critical issues in the representation of disability within Paralympic sport communication, scholars increasingly draw attention to the persistent reliance on stereotyped narratives that emphasize the extraordinary abilities of elite para-athletes with support needs, often framed through the use of advanced technologies such as racing wheelchairs or prosthetic limbs (20, 31). Technocentric innovation has become a defining pillar of the Paralympic Movement, embodied in the figure of the “superhuman” or “supercrip” athlete whose use of carbon-fiber blades, racing wheelchairs, or other cutting-edge devices is celebrated as evidence of exceptional athletic achievement (41). The concept of the supercrip refers to “an individual who through courage, dedication, and hard work accomplishes what is generally seen as the “impossible” in light of the individual's bodily limitations” (46, p. 14). While such high-profile representations can position Paralympic athletes as role models and challenge the ableist bias of mainstream sport, which privileges the finely tuned, able body and marginalizes those who deviate from this norm (47, 48), they are not without problems. Critics argue that the supercrip narrative risks creating unrealistic expectations about the capabilities of people with disabilities and obscures the structural and social barriers they confront in everyday life (49–51).

Moreover, media emphasis on technologically enhanced athletes tends to overshadow other Paralympic sport categories that involve less visible impairments or performances that are less spectacular to able-bodied audiences. This selective focus contributes to a hierarchy of representation within disability sport, where certain bodies and technologies receive disproportionate attention. Extending this critique, Pullen, Mora, and Silk (21, p. 272) highlight a similar trend in the self-representation of female athletes with disabilities on social media, noting how the dominant focus on prosthetics and assistive technologies has given rise to what they define *cripvertising*: a form of commercial communication that entangles disabled subjectivities with “influential brands seeking to align themselves with inclusionary message”.

The prioritized media focus on inspiring disability representation, emphasizing the image of Paralympic athletes as “unfailingly (and even to some extent unrealistically) positive: they are consistently presented as determined, competitive, steely, inspirational” (32, p. 666) has been conceptualized as inspiration porn aimed at engaging and motivating non-disabled people with feel-good life stories of people with disabilities (52). Grue (53, p. 847) defines the inspiration porn construct as “desirable but undesired characteristic”, the one that is positive and inspiring to see, but not personally desired for the spectator, and highlights that the impairment is always visible or symbolically represented with a wheelchair or prosthetics. Furthermore, the excessive attention to the personal impairment of this intentionally inspiring approach often explicitly reinforces the assumption of personal tragedy, reproducing the medical model of disability (54–56), enhancing the difference or otherness of people with disabilities (47, 51) and diverting attention from understanding disability as a personal identity construct (57).

The inspiration porn dimension has been also identified in the context of the Paralympic sponsorship practice. While sporting performance is the main criterion for the Paralympic athletes to access sponsorship deals, Beldame et al. (19) highlight that not all elite Paralympic athletes are equally inspiring to the sponsors, and certain types of disability prevail. Discussing the case of boccia, the authors highlight that the less known or identifiable for non-disabled audience sports categories are usually left behind by sponsors. On contrary, media visibility of superhuman and supercrip elite athletes and their likeability to access sponsorship deals was revealed as significantly higher (19). Similarly, Howe (41) argues that the technocentric ideology of the Paralympic movement makes a technologically enhanced athletic body more valuable than a body with less visible impairments such as visually impaired, athletes with intellectual disability or ambulant cerebral palsy.

While the use of people with disabilities in advertising was found to elicit high emotional and positive reaction to the ad from non-disabled people (58), research by Jang and Kwak (59) reveals that inspiration porn narratives mainly enhance the perception of people of disability as subjects of pity and sympathy. However, the focus on sports achievements of athletes with disabilities elicited high levels of admiration, equality and respect. Furthermore, the perception of pity in inspiration porn narratives was linked to lower levels of sponsor brand authenticity (59).

Discussing the evolution of the media representation of elite Paralympic athletes during the 2014 Glasgow Commonwealth Games, McPherson et al. (32) draw attention also to a celebrity-star approach, framing them as stylish celebrities and iconic models, with higher incidence in the case of female para-athletes. Aimee Mullins has become one of the most popular celebrity icons of this new iconic prosthetic aesthetic representation of disability, showcasing her prosthesis as a symbol of beauty and personal empowerment of her disabled feminine identity (21, 60). The higher prominence of female Paralympic athletes featured with this celebrity-star framing reveals their “glamorised or even eroticized” representation, displacing their parasport identity whereas male elite Paralympians are usually framed as embodiment of strength, stamina, speed, and skills (16, p. 21). This commonly used celebrity approach in advertising communication raises disability scholars' concerns about the prevailing gendered/sexual objectivation and trivialization of Paralympic athletes (21, 32).

The transformative role of digital and social media in Paralympic coverage is also increasingly highlighted by scholars. Antunovic et al. (61) argue that in the context of hegemonic marginalization of Paralympic sports on main broadcasting platforms, digital media have the potential to make a significant contribution to the visibility of disability sport coverage and representation of Paralympic athletes. However, despite the increasing impact on sponsorship opportunities of virtual experience of sports events, research results of Burton, Naraine and Scott (12) reveal that most sponsoring brands lack a consistent online strategy to engage their followers in meaningful narratives of the Paralympic movement, beyond the tactical congratulations for sports achievements and the showcase of national pride.

### 2.3 Paralympic sponsorship framework in Spain: the Spanish Paralympic Committee

The SPC was established in 1995 to unite, coordinate, and represent Spanish Paralympic athletes and teams in close collaboration with the National Sports Council and the national federations governing sports for people with disabilities. Since its inception, the SPC has prioritized both the promotion of Paralympic values and the provision of support to Spanish Paralympic athletes, teams, and sports federations.

Paralympic sponsorship has become a central institutional mechanism for engaging Spanish companies and brands in the Paralympic Movement. As of 2025, the Spanish Paralympic Team is supported by over 20 sponsors, including both global and national brands, through the ADOP Plan (Support to Paralympic Key Sports in Spanish), launched in 2005 and coordinated by the SPC in partnership with the National Sports Council. The ADOP Plan is designed to provide Spanish elite Paralympic athletes with financial aid, administrative assistance, specialized healthcare insurance, and an interdisciplinary support team, including physiotherapists, sports nutritionists, psychologists, and coaches, to ensure optimal conditions for training and preparation for the Paralympic Games. In return, sponsors receive image endorsement from the SPC as well as a series of corporate tax incentives. Sponsorship initiatives within this framework include personal grants for athletes, financial support for Paralympic sports federations, and communication and promotional campaigns aimed at raising awareness about the Paralympic movement.

The regulatory context further reinforces this commitment. The Spanish Sports Act 39/2022 (62) declares of general interest the social inclusion of persons with disabilities through sports participation and the promotion and practice of inclusive sport, defined as joint sporting activities between people with and without disabilities (Article 6), and mandates the effective inclusion and representation of athletes with disabilities within national sports federations (Article 47). In addition, the Act underscores the importance of enhancing media visibility for both inclusive and disability sports more broadly (Article 6).

## 3 Materials and method

The purpose of this exploratory study is to review the historical evolution of Sanitas' Paralympic sponsorship in order to identify and systematize its key strategic underpinnings, main action lines, and relevant milestones. The findings aim to contribute to Paralympic scholarship by revealing both the critical challenges and emerging opportunities of Paralympic sponsorship, while also providing broader insights into the complexities of para-sport communication. At the same time, the study seeks to generate actionable knowledge that can be used by brand managers, Paralympic athletes, teams, and sports federations to optimize their sponsorship strategies.

A case study approach, conceived as an empirical inquiry and in-depth analysis of a contemporary phenomenon, guided the data collection and analysis (63). Secondary data were collected through desk research and monitoring of digital platforms managed by the SPC, IPC, Sanitas Foundation, and BUPA, which provided a historical overview of Sanitas' Paralympic sponsorship. Primary data were generated through a semi-structured, in-person interview conducted by the first and second authors with the Chief Sustainability and Corporate Affairs Officer of Sanitas, who also serves as the director of the Sanitas Foundation. The interviewee was selected due to their central role in developing the sponsorship agreement since 2007, their leadership in advancing disability and inclusion, and their expertise in transferring Sanitas’ sponsorship practices internationally within the BUPA Group. Informed by existing scholarship on Paralympic sponsorship, the interview was conducted by the first and second authors at Sanitas’ headquarters in Madrid on May 20, 2024. It explored Sanitas' long-standing partnership with the SPC and the evolution of its model from a traditional sponsorship approach to a holistic framework that promotes inclusion and accessibility. The interview was transcribed, checked for accuracy, and translated into English for analysis. Informed consent was obtained from the participant, including agreement for the interview to be recorded (64). Research data were analyzed using reflexive thematic analysis (65).

The analysis was further enriched by the insider research perspectives of two authors with professional engagement in the case under study. The second author has led the corporate communications area of the SPC since 2005, while the third author has directed the Sanitas Foundation Chair for Inclusive Sports Studies since 2017. These dual roles as practitioners and researchers provided access to tacit knowledge, insider perspectives, and nuanced understandings of the everyday practices and challenges within Sanitas' Paralympic sponsorship. This positionality enhanced the depth of analysis by allowing the team to identify contextually significant dynamics that might remain invisible to external observers (66, 67). At the same time, we recognize that such proximity carries risks of bias. To mitigate this, the research team engaged in reflexive dialogue and relied on the perspective of the first author, who was not directly involved in the Sanitas sponsorship activities, to critically interrogate assumptions and interpretations.

## 4 Sanitas' Paralympic sponsorship journey: from partnership to inclusion

### 4.1 From healthcare assistance to athletes with disabilities to leading multi-stakeholder engagement in the Paralympic Movement

Sanitas' interest in supporting the Paralympic movement emerged at the beginning of 2002 when Sanitas signed an official sponsorship agreement with Real Madrid's football and basketball teams to provide their athletes with exclusive medical assistance (68). At the same time, the company was also delivering medical support to the Spanish National Organization of Blind People (ONCE) (69). These two partnerships, one with Spain's most prominent sports brand and the other with a leading organization promoting the inclusion of people with disability, paved the way for Sanitas’ first agreement with the SPC in 2007, when it became the Official Health Partner of the SPC (70).

The initial agreement established two main areas of collaboration. First, Sanitas assumed the role of official sponsor of the Spanish Paralympic Team, providing direct financial support to athletes. Second, a healthcare framework was created to ensure comprehensive medical coverage for elite Paralympic athletes in Spain. This included: (a) exclusive medical and physiotherapy staff contracted by the SPC but based in a fully serviced Sanitas hospital in Madrid; (b) healthcare insurance policies for all leading Paralympic athletes; and (c) the deployment of *Medical Clinic Spain* by Sanitas within the Paralympic Village during the Games. Erburu (71) notes that no comparable model of healthcare provision had previously existed for a national Paralympic team in Spain, positioning Sanitas as a pioneer in Paralympic healthcare assistance.

One of the outcomes of the Sanitas partnership with the SPC was the launch in 2008 of *Sanitas Inclusive*, an innovative healthcare insurance designed specifically for people with disabilities in Spain. This service was developed in collaboration with the National Organization of Blind People and the SPC, which helped identify priority areas of medical assistance and establish accessibility guidelines for hospitals delivering this specialized care. Prior to 2008, no private insurance company in Spain had provided healthcare coverage tailored to people with disabilities. Although the number of insured individuals remains modest, Erburu (71) highlights its positive impact on users' well-being and frames it as a further demonstration of Sanitas' long-term commitment to social inclusion.

Soon after, the partnership expanded to focus more explicitly on promoting inclusive sports for people with disabilities on a broader scale. What began as a traditional sponsorship arrangement, whereby a brand contributes financially to a sporting property in exchange for endorsement and reputational benefits (4), gradually evolved into a distinctive ambition to foster an inclusive sports culture in Spain (71). Reflecting this shift, the Sanitas Foundation designated the promotion of inclusive sport as a strategic priority. In 2009, it founded the Research Center for Inclusive Sports at Universidad Politécnica de Madrid (72), which in 2017 became the Sanitas Foundation Chair for Inclusive Sports Studies (73). Inclusive sport is defined as the joint practice of sport by people with and without disabilities, sharing the same facilities and spaces.

In 2010, the Sanitas Foundation launched the *Strategic Alliance for Inclusive Sport*, an initiative designed to transform the disability paradigm in sport by promoting the visibility and social acceptance of inclusive sport. Its objective was to enable joint participation in training and competition for people with and without disabilities across all age groups, with particular emphasis on engaging younger generations. The founding partners of the Alliance included the Spanish Olympic Committee, the SPC, Madrid City Council, the Autonomous Community of Madrid, the Real Madrid Foundation, the National Organization of Blind People, the Spanish National Broadcasting Corporation, and Madrid's regional television channel. The initiative also received strong support from civil society organizations and sports federations committed to advancing the normalization of inclusive sport (74). This innovative emphasis on fostering an inclusive sporting mindset was aimed at enhancing visibility of the challenges faced by Paralympic sport, creating synergies between public institutions, sports federations, and civil society, and placing inclusive sport on the agendas of policymakers, educational institutions, and the broader public. As part of its advocacy, the partners of the Strategic Alliance launched the *Manifesto for Inclusive Sport* (74), which emphasizes the importance of embedding inclusion as a core value in sport.

### 4.2 The focus on inclusive sports practice and values

Building on the multi-stakeholder synergies generated by the *Strategic Alliance for Inclusive Sport*, the Sanitas Foundation launched three flagship initiatives: the *Inclusive Sport at School* educational program, *Sanitas Inclusive Week*, and the *Sanitas Inclusive Games*.

The *Inclusive Sport at School* program has been running by Sanitas Foundation since 2012 with the purpose to provide training methods and academic resources of Paralympic sports to physical education schoolteachers to help them enable an effective inclusion of children with disabilities in physical education activities (75, 76). The program is based on three main psychological strategies: contact theory, allowing a Paralympic athlete to visit the school and share with students their experience with sports; information theory, providing access to resources and materials enabling teachers and students to discover the basics of sport for people with disabilities with guides, videos and tasks; and simulation theory by offering practical exercises simulating a physical, sensorial or intellectual disability. Grassi-Roig et al. (77) highlight that this educational initiative has become a successful awareness program about inclusive sports practice among schoolchildren.

It is also noteworthy that, in 2018, the Spanish National Sports Council and several national sports federations approved the participation of school-age athletes with disabilities in the Spanish Championships for Autonomous Community Teams. This landmark decision marked the first official inclusive competition in Spain. The event was widely praised by national federations for its contribution to promoting inclusion values in sport, increasing visibility and awareness of disability, and highlighting the urgent need to expand competition opportunities for people with disabilities, given the traditionally limited number of events available to them (78). Reflecting on the initiative, Erburu (71) noted: “We have created something new and have served as a catalyst for social transformation, which we believe is the work of the Sanitas Foundation, beyond just making donations”.

The *Inclusive Sport Week* is another relevant initiative closely aligned with the *Strategic Alliance for Inclusive Sport* activities. Since 2010, the *Inclusive Sports Week* has been held annually each October at Universidad Politécnica de Madrid, combining academic and scientific sessions with initiatives designed to promote the practice and visibility of inclusive sport (79). The event engages a wide range of stakeholders, including students, researchers, athletes, coaches, and sports managers. Activities range from academic debates addressing the critical challenges of sport for people with disabilities, to outdoor practice of inclusive disciplines, particularly those not widely developed at the national level, such as wheelchair rugby (80). The program also incorporates high-profile events, such as the Guinness World Record for the largest indoor cycling activity, achieved during the 7th Inclusive Sports Week in 2016, when 1,200 participants gathered in Madrid's Plaza Colón. In 2025, the program reached a new milestone with the *All Star Inclusive Basketball Match*, the first global event of its kind to bring together leading Spanish players and US NBA stars in two mixed teams of athletes with and without disabilities. The match took place in one of Madrid's most iconic sports arenas and was attended by Sanitas employees, schoolchildren, top Spanish and international basketball players, and the Mayor of Madrid (81).

The consistent emphasis on the promotion of inclusive sports practice and values as a premise of the effective normalization of sports equity and diversity, led Sanitas together with the *Strategic Alliance for Inclusive Sport* to launch in 2021 the first edition of the *Inclusive Games*. The planning process unfolded in three phases: the identification of national sports federations interested in joining the initiative; the development of specific regulations for each discipline; and, ultimately, the selection of participating athletes. Competition formats were built around five guiding principles: safety, to guarantee the integrity of all athletes, particularly in disciplines requiring joint participation of people with and without reduced mobility; adaptation, ensuring accessibility through modifications to equipment and spaces; fair contribution, so that every athlete's performance equally impacted team outcomes regardless of disability; respect for the essence of the sport, maintaining competitive integrity without limiting athletes without disabilities; and full participation, guaranteeing equal opportunities and conditions for all competitors (82). The event brought together 167 elite Spanish athletes with and without disabilities to compete across nine disciplines: athletics (*n* = 63), badminton (*n* = 4), judo (*n* = 9), rugby (*n* = 18), swimming (*n* = 34), table tennis (*n* = 8), taekwondo (*n* = 7), triathlon (*n* = 7), and wheelchair basketball (*n* = 17). The Games were initially scheduled for 2020 but were postponed to October 2021 due to the Covid-19 pandemic. Participants included Olympic and Paralympic athletes who had competed at the Tokyo 2020 Games.

The second edition of the *Inclusive Games* was held in Madrid in 2024, expanding to 14 sports and involving 260 Olympic and Paralympic athletes: archery (*n* = 12), athletics (*n* = 64), badminton (*n* = 4), fencing (*n* = 6), field hockey (*n* = 32), judo (*n* = 8), karate (*n* = 24), rugby (*n* = 16), swimming (*n* = 32), table tennis (*n* = 12), taekwondo (*n* = 4), triathlon (*n* = 8), wheelchair basketball (*n* = 20) and wheelchair handball (*n* = 18). For the first time, international competitors joined from Chile (*n* = 2), Ecuador (*n* = 1), India (*n* = 1), Italy (*n* = 1), Mexico (*n* = 2), Poland (*n* = 1), Spain (*n* = 247), and the United Kingdom (*n* = 5), further enhancing the event's global visibility and impact (83). Erburu (71) describes the *Inclusive Games* as a natural evolution of the Olympic and Paralympic movements, highlighting their catalytic impact in fostering collaboration across institutional and sporting agencies: “We knew that high-level competition was the most challenging, as each has its own rules, and organizing the competitions and regulations was necessary for both worlds to compete together. That's when we truly saw how incredible this is, because no one said no: the sports and disability federations, the Olympic federations all came together to design the competitions, bring in referees, judges, facilities … it was amazing”.

### 4.3 Inclusive communication strategy

From the beginning of its partnerships with both the SPC and Spanish Olympic Committee, Sanitas has consistently pursued an inclusive communication strategy. In 2019, as an extension of its partnerships with both the SPC and the SOC, Sanitas launched the *Sanitas Team*, an inclusive group of brand ambassador athletes with and without disabilities. The team currently includes six elite Spanish athletes: Paralympians Sara Andrés (athletics) and Eva Moral (triathlon), alongside Olympians Carolina Marín (badminton), Sergio Llull (basketball), Saúl Craviotto (canoeing), and Chema Martínez (athletics). All of Sanitas Olympic and Paralympic Games campaigns have featured athletes from both teams, a practice that Erburu (71) describes as one of the company's proudest achievements: “When we plan a campaign, whether it is a Christmas social media activation or an event with the Sanitas Team, our aim is always to feature them together, Olympic and Paralympic athletes”. The inclusive team of Sanitas ambassadors is involved in all relevant projects launched by the company, such as the *Healthy Cities* project, a sustainability initiative promoting physical activity and healthy lifestyles in urban areas. The program encourages participants to walk 6,000 steps daily and to avoid using combustion vehicles at least one day per week. The *Sanitas Team* leads the community walks. More than 63,000 people participated in recent editions of *Healthy Cities* (84).

Another relevant milestone of the multifaceted approach to Paralympic sponsorship and the promotion of inclusive sports mindset of Sanitas is a documentary series *Power* in Amazon Prime featuring personal and sports profiles of top Spanish Paralympic Athletes and Sanitas brand ambassadors (85). The three episodes of *Pow*er produced by Sanitas Foundation present the daily sports and life experience of a male wheelchair basketball player, a female track and field athlete, a female para-swimmer and a male para-badminton player. Their experience in para-sports intensive training and competition, family, friends, personal challenges and dreams is the main narrative of the series, presenting their identity as elite athletes and people with disabilities. In parallel, each episode features the inclusive sports experience in each of the sports categories of the athletes. Top Spanish and World basketball, badminton, canoeing, swimming, and track and field champions join the Paralympic athletes in inclusive sports practice. The *Power* series became a trending topic on social media with #powerinclusion hashtag.

The Paralympic sponsorship and communication model pioneered by Sanitas in Spain was subsequently transferred to three other countries, Poland (2014), Chile (2020), and the United Kingdom (2022), through the national branches of BUPA, the parent company of Sanitas. In each case, BUPA became the *Official Healthcare Partner* of the respective national Paralympic Associations (70, 86). In the lead-up to the Paris 2024 Games, the model expanded further with new Paralympic partnerships established in Australia, New Zealand, and Mexico. Erburu (71) emphasizes that the Spanish model, which moved well beyond the traditional sponsorship logic of financial contributions in exchange for image rights, proved instrumental in shaping and guiding all new Paralympic partnerships undertaken by BUPA. Furthermore, before the Paris 2024 Games, BUPA launched a global communication campaign *Picture of Health* shoot by Annie Leibovitz featuring six international Paralympic athletes sponsored by BUPA and Sanitas (87).

### 4.4 The engagement of Sanitas employees in the Paralympic sponsorship initiatives

A distinctive feature of Sanitas' Paralympic Sponsorship strategy is its sustained emphasis on employee engagement. Key sponsorship milestones are actively disseminated through internal communication channels, while employees are directly involved in initiatives such as the *Inclusive Games*, *Inclusive Sports Week*, *Healthy Cities*, and events with Spanish Paralympic athletes. According to Erburu (71), the sponsorship enjoys strong internal support, fostering a sense of pride and belonging among staff: “It's something beyond what a classic sponsorship is, putting out the logo, running a campaign, and putting up a photo. There is a higher mission. It is something that is about values. And in the company, I believe it is appreciated for that. I think it makes us better”. One of the most memorable corporate moments, she notes, occurs when Paralympic athletes visit Sanitas headquarters upon their return from the Paralympic Games to celebrate and meet employees. Inclusive sports activities are also embedded into the company's culture, frequently incorporated into team-building initiatives. Overall, Paralympic Sponsorship is regarded as one of the most valued corporate programs among Sanitas employees.

## 5 Discussion and conclusions

The Paralympic movement, and Paralympic sponsorship in particular, continue to face significant challenges, shaped by the power imbalance between the IOC and the IPC and the subordinate status of the Paralympic Games (23). These challenges are compounded by the global invisibility of Paralympic sport and the persistence of stereotypical representations of people with disabilities in media narratives (29–31, 88). For brands, achieving an objective and respectful representation of disability, without falling into reductive frames or simplistic stereotypes, remains a critical challenge when aligning with values of diversity and inclusion through Paralympic sponsorship (32).

Although the number of Paralympic sponsors remains limited globally, emerging innovative approaches are expanding the boundaries of traditional sponsorship by creating meaningful experiences of corporate engagement with the Paralympic movement (5). Grounded in the values of diversity, equity, and inclusion, these approaches redefine sponsorship as more than a transactional exchange of financial resources for image rights. Instead, sponsorship becomes a catalyst for social transformation with the potential to shift cultural perceptions of disability.

This study has offered an in-depth analysis of the Paralympic sponsorship strategy of Sanitas, a Spanish healthcare insurance company and part of the global BUPA group. The review of Sanitas’ long-term partnership with the SPC illustrates a consistent, strategically grounded approach to Paralympic sponsorship, while also providing actionable insights for brand managers, Paralympic teams, and athletes navigating this promising but complex sponsorship landscape. The historical evolution of Sanitas' involvement with the SPC shows a transformative journey from an initial focus on financial and healthcare support, to the creation of multi-stakeholder alliances and the development of ambitious initiatives aimed at fostering an inclusive social mindset.

The key strategic dimensions of this transformative model can be summarized as follows:

A strong emphasis on inclusive sports education for younger generations through dedicated school and university programs.
Recognition of the social relevance of inclusive sports practice at both professional and amateur levels, ensuring broad stakeholder engagement and cross-sectoral synergies.
The organization of impactful sporting and social events featuring elite Olympic and Paralympic athletes, generating high media visibility and lasting social legacies.
The development of innovative visual narratives that communicate the multifaceted ecosystem of Paralympic sponsorship.
Active involvement of employees, embedding Paralympic sponsorship as a core transformative value within the corporate culture.
Internationalization of Paralympic sponsorship expertise and transfer of best practices.

Taken together, these dimensions illustrate an innovative Paralympic sponsorship approach of Sanitas aimed at providing support to the Paralympic movement, while also reshaping cultural understandings of disability and inclusion.

As Hardin and Billings (20) remind us, visibility alone is not enough to break the barriers that hinder the full inclusion of athletes with disabilities in sport and society. Through its innovative and disruptive approach by challenging the traditional separation of athletes with and without disabilities and creating a shared competitive space, Sanitas aims to redefine the meaning of inclusion in sport, positioning the Paralympic sponsorship as a driver of social transformation.

## 6 Limitations

For this study, we adopted an exploratory and interpretive case-study approach to examine the historical evolution, key milestones, and best practices of the Paralympic Sponsorship model developed by Sanitas since 2002. Our analysis focused specifically on the strategic decisions made by the Sanitas leadership team, with the purpose of providing a comprehensive though not exhaustive overview of the main dimensions, actions, and narratives underpinning the company's Paralympic Sponsorship strategy. Data were gathered through extensive desk research and an interview with the Sanitas Chief Sustainability and Corporate Affairs officer and General Manager of the Sanitas Foundation. Although conducting a single interview may be viewed as a potential limitation, the interviewee's senior corporate position at Sanitas and their direct involvement in the decision-making processes related to the sponsorship strategy provide a strong rationale for this methodological choice. Future research could build on our findings by engaging additional stakeholders within the Paralympic sponsorship ecosystem (including the SPC, Spanish sports federations, and Paralympic athletes) to further assess the impact of Sanitas' sponsorship initiatives and identify remaining challenges. Additionally, while the insider perspective of two authors with professional roles connected to the case may also be considered a limitation, their access to tacit knowledge and nuanced insights into Sanitas' sponsorship practices was considered essential to the development of this case study.

## Data Availability

The raw data supporting the conclusions of this article will be made available by the authors, without undue reservation.
